# Supplementary figures and images for: Responses of Escherichia coli and Listeria monocytogenes to ozone treatment on non-host tomato: Efficacy of intervention and evidence of induced acclimation
Source: PLoS One. 2021 Oct 28;16(10):e0256324. doi: 10.1371/journal.pone.0256324 (PMC8553054; doi:10.1371/journal.pone.0256324)

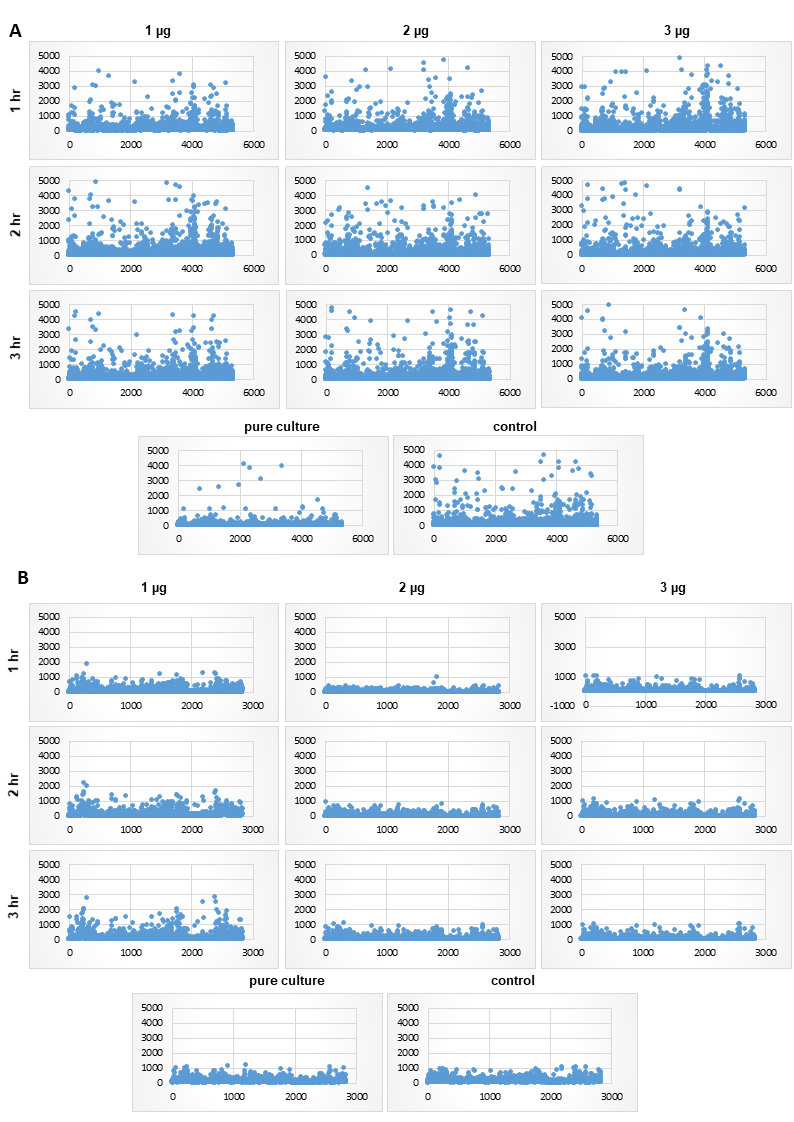

Supplement: S1 Fig — Scatter plots of the RPKM values for each transcriptome library of E. coli (A) and L. monocytogenes (B) in response to 1, 2 and 3 μg of O3 per gram of ripen fruits at 1 hour (hr), 2 hr and 3 hr, respectively, including the control and pure culture. (TIF) [file pone.0256324.s001.tif]
